# Fluoride mouthrinses for prevention of initial caries in orthodontic patients - a systematic review and meta-analysis

**DOI:** 10.1186/s12903-025-06374-8

**Published:** 2025-07-02

**Authors:** Mikael Sonesson, Svante Twetman

**Affiliations:** 1 Department of Orthodontics, Faculty of Odontology, Malmö University, Carl Gustavs väg 34, Malmö, SE-205 06 Sweden; 2 Department of Odontology, Faculty of Health and Medical Sciences, University of Copenhagen, Copenhagen, Denmark; 3 Department of Pediatric Oral Health and Orthodontics, University Center for Dental Medicine Basel UZB, University of Basel, Basel, Switzerland

**Keywords:** Caries, Enamel demineralization, Fluoride, Mouthrinse, Orthodontics, White spot lesion

## Abstract

**Background:**

Orthodontic patients are often instructed to use fluoride mouthrinses (FMR) to prevent caries during treatment with fixed orthodontic appliances (FOA). The aim of this study was to examine the caries preventive effect of FMR during FOA treatment based on randomized controlled trials.

**Methods:**

An information specialist searched five databases up to September 30, 2024. We included trials with parallel groups (intervention vs. control) and a minimum duration of six months. Based on the abstracts, the authors independently selected and reviewed full text papers, extracted key outcome data, and assessed the risk of bias. The primary outcome was incidence of enamel caries on subject level. We conducted a narrative synthesis and pooled comparable data in a random effects model.

**Results:**

We identified 22 studies of which seven, involving 704 patients, met the inclusion criteria. Five and two studies had moderate and high risk of bias, respectively. In all studies, FMR was additive to daily use of fluoride toothpaste. The intervention varied from twice daily to twice weekly and the duration ranged from six to 26 months. Five studies were included in a meta-analysis. The aggregated data showed a small risk difference of − 0.07 (95% CI -0.14; -0.01) in initial caries development adjacent to bracket base between the experimental and the control groups.

**Conclusion:**

This review found insufficient support for a general recommendation to use FMR during treatment with fixed orthodontic appliances in populations with regular use of fluoride toothpaste. This does not rule out the possibility that individual orthodontic patients may benefit from FMR after comprehensive risk assessment. Further investigations with standardized interventions and duration, reporting a core outcome set are required to clarify the effectiveness of fluoride mouthrinses in orthodontic patients.

**Supplementary Information:**

The online version contains supplementary material available at 10.1186/s12903-025-06374-8.

## Introduction

Orthodontic treatment with fixed appliances is associated with an increased caries risk [1] and the incidence of enamel demineralization and white spot lesions in subjects undergoing such treatment is high, representing a challenge for both patients and dental professionals [2]. Therefore, enhanced caries preventive strategies need to be implemented, and systematic reviews have displayed moderate certainty evidence that topical fluorides, such as fluoride mouthrinses, can reduce caries increment in young permanent teeth, in particular among those with a limited background fluoride exposure from toothpaste [3, 4]. Therefore, in addition to thorough oral hygiene instructions, orthodontists often recommend daily or weekly fluoride mouthrinses (FMR) for their patients with fixed appliances [5–9]. The effectiveness has however been questioned as the certainty of available evidence was graded as low or very low [10, 11]. Obviously, the effect of FMR is strongly dependent on the patient compliance and according to Geiger and co-workers [12], less than 15% of the orthodontic patients used FMR on a daily basis as instructed. Today, novel fluoride compositions and elevated fluoride concentrations are available and targeted to orthodontic patients [13] and this merits an updated compilation of clinical trials concerning the effects of FMR in the orthodontic setting. The aim of this study was therefor to examine the caries preventive effect of fluoride mouth rinses during treatment with fixed orthodontic appliances based on randomized controlled trials. The specific research question was “Do fluoride mouthrinses help to prevent initial caries during orthodontic treatment with fixed appliances?”

## Methods

The present systematic review was reported in accordance with the checklist of preferred reporting items for systematic reviews and meta-analyses (PRISMA) and the study protocol was registered at PROSPERO (CRD42024583520). We searched for relevant literature with the help of the following PICO:

*Population* – Healthy children, adolescents and adults undergoing orthodontic multi-bracket treatment.

*Intervention* – Regular (daily to weekly) fluoride mouthrinses during the course of orthodontic treatment with a duration of at least 6 months.

*Control* – Placebo mouthrinses, any non-fluoride mouthrinse/mouthwash, other non-fluoride comparisons or standard care (treatment as usual).

*Outcome* – (i) Incidence of white spot lesions adjacent to brackets, at least 6 months after onset of appliances or within 6 months after debonding, assessed with a clinically defined demineralization index, and (ii) Incidence of proximal caries lesions scored from bitewing radiographs.

### Search and study retrieval

An information specialist at Malmö University, Sweden, searched the electronic databases PubMed, Scopus, Web of Science and Cochrane from January 1985 up to September 30, 2024 for relevant literature. We also consulted Google Scholar for additional information. We provide the detailed search terms in Supplementary file A. The eligibility criteria were peer-reviewed publications in English, reporting a clinical intervention in patients undergoing treatment with fixed orthodontic appliances. We did not consider grey literature, dissertations or conference abstracts. We requested categorized or continuous data assessed clinically or from digital photos/radiographs reported on patient level. Papers reporting continuous data were not included in the meta-analysis. The reference lists of all selected papers, including available systematic reviews, were hand-searched for possible additional references. We searched www.clinicaltrials.gov to identify registered ongoing studies by combining the phrase “fluoride mouthrinse/mouthwash” with “orthodontics” and/or “fixed appliances”.

### Selection of studies and data extraction

**Fig. 1:**
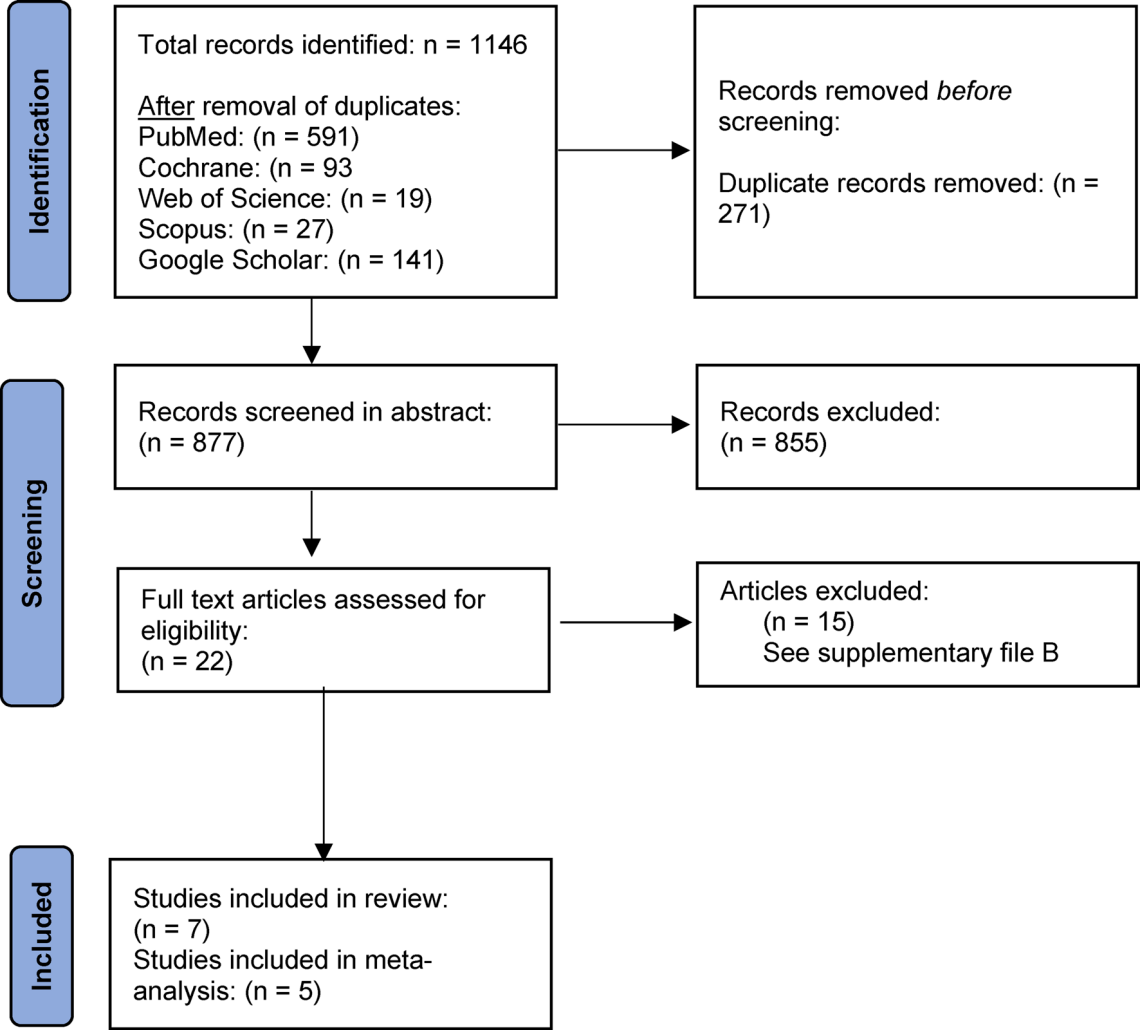
PRISMA flowchart displaying the search and selection process

The search process gave 1,146 hits and 877 remained after removal of duplicates. The two authors assessed the abstracts of potentially eligible studies independently and ordered full-text papers for further evaluation of relevance (Fig. 1). Disagreements were resolved in consensus. The two authors extracted data from the included studies independently from each other. We tabulated the following items: first author, year of publication, country of origin, age of study group, duration of intervention, dropout rate and compliance. In addition, the authors listed the interventions in the test- and control groups together with the number of subjects that completed the trial. The primary outcome measure was the incidence of enamel caries on subject level. The data was dichotomized into “no incidence of demineralization” vs. “any demineralization” on subject level. In two cases, we contacted the corresponding author by e-mail for clarifications.

### Assessment of risk of bias

The authors assessed the risk of bias (low, moderate, high) independently for each study according to the Cochrane Handbook for Systematic Reviews of Interventions [14]. We solved disagreements with a consensus discussion.

### Data synthesis

The authors conducted a narrative synthesis of the included studies. For studies with comparable outcome measures, we pooled data in a random effects model using the Review Manager 5.3 tool (The Nordic Cochrane Centre, Copenhagen, Denmark).

## Results

We identified 22 papers published between 1988 and 2024, of which 15 were excluded after full text scrutiny (Supplementary file B). We show the main characteristics of the remaining seven studies in Table 1A and 1B [15–21]. The studies were conducted in Sweden, Denmark, The Netherlands, India, Iraq and Pakistan, involving a total number of 704 adolescents and young adults. Two articles originated from the same study population [18, 19] but reported different endpoints. The dropout rates ranged from zero to 32.5%. All studies were randomized controlled trials; one was single-blind [19], two were double-blind [16, 17] and two were triple-blind [15, 20]. In one study, the outcome was assessed by non-blinded examiners [21]. Two trials applied a placebo-controlled design [15, 20]. Five studies had an average duration of ≥12 months while two had their final follow-up six months after the onset of appliances [16, 17]. The fluoride mouthrinses under study contained 0.05–0.32% sodium fluoride, applied once to two times daily, or twice weekly [17–21], amino fluoride in one study [16], and a mix of amino fluoride and sodium fluoride in one study [15]. The endpoint was assessed with clinical indices according to Gorelick or IDCAS, either through visual inspection or from digital photos, in five studies [16, 17, 19–21] while two studies relied on QLF [15] or bitewing radiographs [18]. In all studies however, two times daily toothbrushing with fluoride toothpaste (1100–1450 ppm) was emphasized for all participants.

### Risk of bias within studies

Table 2 displays the risk of bias of the included studies. We assessed five studies at moderate risk of bias [15, 17–20] and two with high risk of bias [16, 21].

**Table 1A:** Main characteristics of the identified studies

First author, year; country/study design	Age	Duration	Dropout/ compliance	WSL endpoint
Ali, 2022; IRQ/RCT	18-37 yrs.	6 mo	0%/NR	ICDAS, visual, upper and lower anterior teeth
Ekstrand, 2023; DK/RCT	10-12 yrs.	12 mo	13%/NR	ICDAS and bitewing, all teeth with fixed appliances
Enerbäck, 2022; SWE/RCT	12-20 yrs.	23-24 mo	5.6%/good	ΔDiFS (WHO) from bitewing radiographs at debond
Enerbäck, 2023; SWE/RCT	mean ≈15 yrs.	average 26 mo	8.1%/93.8%	Gorelick index from digital photos at debond, all teeth/anterior teeth
Ravikiran, 2021; IND/RCT	12-28 yrs.	6 mo	0%/NR	with vs. without WSL from digital photos, brackets in situ, upper anterior teeth
van der Kaaij, 2015; NL/RCT	mean ≈13 yrs.	average 25 mo	32.5%/NR	QLF, 6 weeks after debond, all teeth
Zahid, 2024; PK/RCT	mean ≈17 yrs.	NR	0%/NR	Gorelick index from digital photos at debond, all teeth

Abbreviations: RCT = randomized controlled trial; NR = not reported

^a^ including banded first permanent molars

**Table 1B:** Interventions and outcome

First author, year	Intervention (*n*)	Control (*n*)	WSL/initial caries incidence
Ali, 2022	0.05% NaF MR; twice daily+FTP (*n*=14)	CHX MR+FTP (*n*=14)	24.4% vs. 24.4% (NS)
Boyd, 1992	0.05% NaF MR; daily (*n*=26)	FTP (*n*=32)	10.1% vs. 14.4%^a^ (NS)
Ekstrand, 2023	0.32% NaF MR, twice weekly+FTP (*n*=30)	Placebo MR twice weekly+FTP (*n*=31)	23.1% vs. 40.7% (S)
Enerbäck, 2022	0.2% NaF MR; twice daily+FTP (*n*=87)	FTP (*n*=90)	39.1% vs. 28.7%^c^ (NS)
Enerbäck, 2023	0.2% NaF MR, twice daily+FTP (*n*=81)	FTP (*n*=82)	65.4% vs. 73.2% (NS)
Ravikiran, 2021	0.1% AmF MR; daily+FTP (*n*=25)	FTP (*n*=25)	0.46 vs. -0.55^b^ (S)
van der Kaaij, 2015	0.05% AmF/NaF MR; daily+FTP (*n*=36)	Placebo MR daily+FTP (*n*=45)	30.6% vs. 46.7% (S)
Zahid, 2024	0.2% NaF MR; twice daily+FTP (*n*=83)	FTP (*n*=83)	66.3% vs. 69.9% (NS)

Abbreviations: MR = mouthrinse; NaF = sodium fluoride; AmF = amine fluoride; SnF2 = stannous fluoride; FTP = twice daily fluoride toothpaste (1100–1450 ppm); WSL = white spot lesion

^a^ percentage of sites

^b^ new initial caries lesions during treatment

^c^ post-treatment mean lesion area; a negative value indicates increase of lesion

**Table 2:**
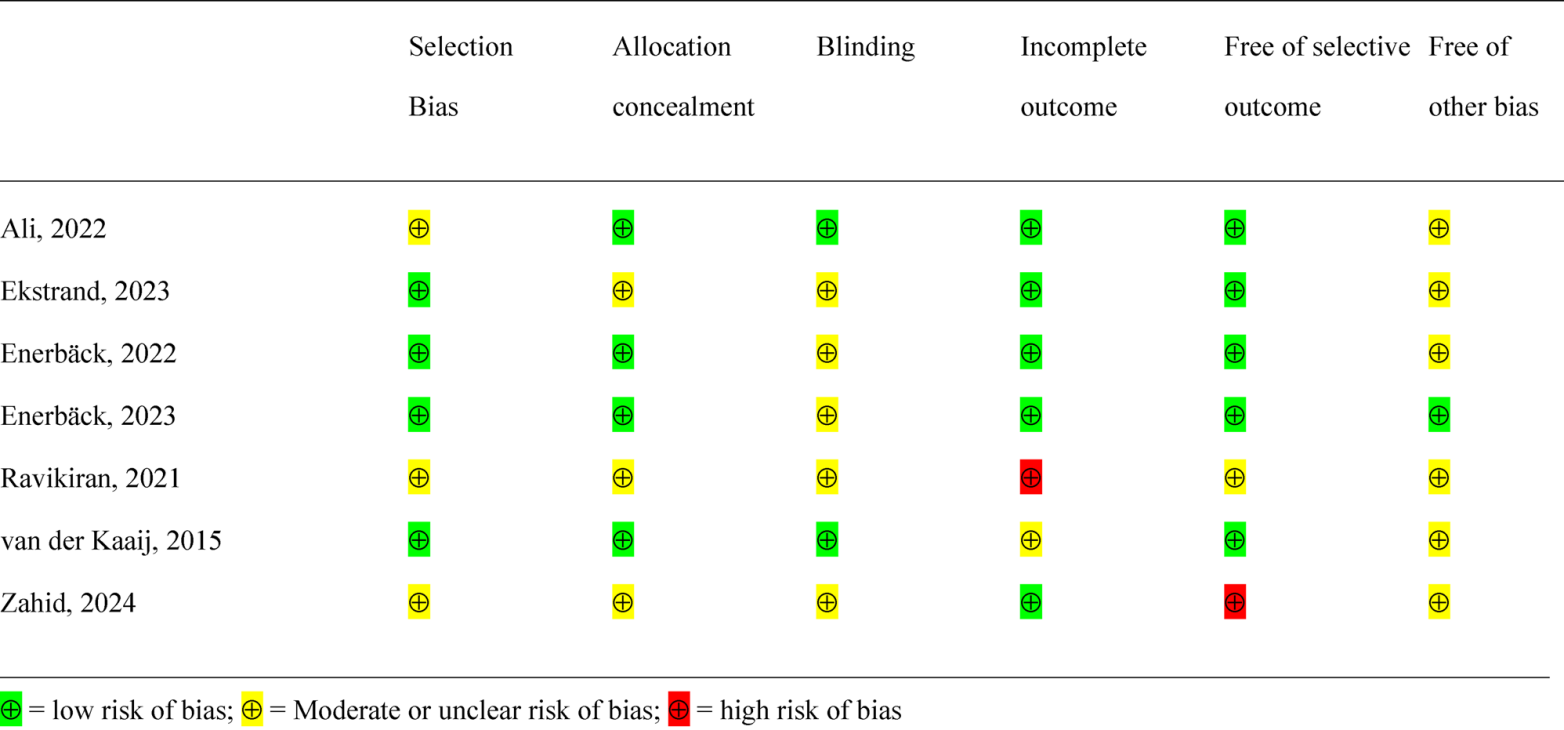
Risk of bias assessment

## Results of individual studies

Three studies [15, 16, 20] presented statistically significant results in favor of FMR plus fluoride toothpaste versus fluoride toothpaste alone (Table 1B). The remaining trials found no significant benefits of FMR on initial caries or white spot lesion development. However, in two studies, a significant reduction of the incidence of initial caries around orthodontic brackets was reported in the FMR group when only teeth in the “aesthetic zone” (central and lateral incisors, canines) were accounted for [19, 21].

No side- or adverse effects were reported in any of the included reports. Due to the limited number of eligible studies, their quality and the inconsistent results, we consider the evidence as inconclusive.

### Meta-analysis

**Fig. 2:**
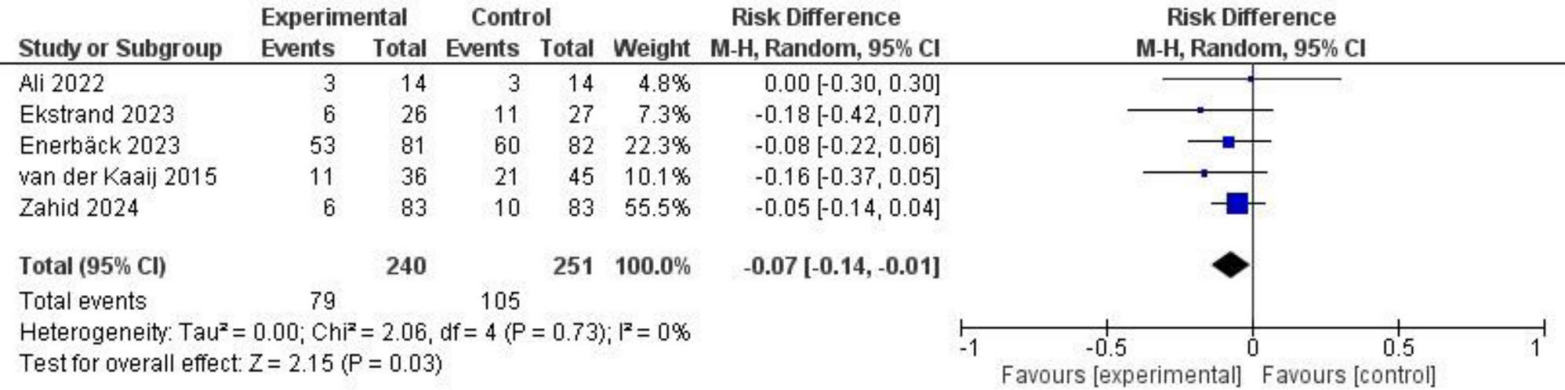
Forest plot showing the effect of fluoride mouthrinse (experimental) on the incidence of enamel caries adjacent to bracket base during treatment with fixed orthodontic appliances vs. control

Five studies based on 491 subjects presented caries incidence adjacent to the bracket base on patient level [15, 17, 19–21] were included in the meta-analysis (Fig. 2). One study presented only mean values of the lesion area [16] and one reported interdental caries incidence from a common study population [18]. The duration of the fluoride mouthrinse intervention ranged from six to 26 months and the time-point of endpoint scoring varied from brackets in situ after six months of intervention to 6 weeks after debonding. The aggregated data showed a risk difference of -0.07 (95% CI -0.14; -0.01) in initial caries development between the experimental and the control groups. The I^2^-value indicated a low heterogeneity (Fig. 2).

## Discussion

Caries development during orthodontic treatment with fixed appliances is an unwanted adverse effect that can jeopardize the esthetic result as well as dental health. Therefore, an elevated fluoride exposure is desirable and regular fluoride mouthrinse (FMR) is one of the available options [6, 22] and there are commercial rinses specially developed and marketed to orthodontic patients [16, 23]. In the present review, we included seven clinical trials on the effect of FMR on the incidence of enamel caries during treatment with fixed appliances. In all trials, the intervention was combined with daily brushing with fluoride toothpaste; this is required for ethical reasons as fluoride toothpaste is listed as an “essential dental medicine” by WHO [24]. Due to the limited number of eligible randomized controlled trials and the inconsistent findings, we found inconclusive evidence to support a general recommendation to use FMR during treatment of fixed orthodontic appliances. However, unreliable evidence is not the same as lack of effect. The aggregated data demonstrated a small difference in favor of FMR, which indicates that the intervention may be beneficial on individual indications after a comprehensive risk assessment. This includes orthodontic patients with elevated caries risk in first hand but also those with gingivitis or halitosis may be considered.

The I^2^-value obtained in the meta-analysis indicated no study heterogeneity but due to the small number of eligible studies, this value must be interpreted cautiously. Likewise, the low number of reports did not allow for an analysis of publication bias. Common quality shortcomings of the included studies were lack of power and double blinding, selection bias and incomplete reporting. Due to the limited number of eligible papers with sufficient sample size, we were unable to perform sub-analysis to evaluate if the fluoride concentration, fluoride formulation, flavor or frequency of rinsing could have an impact on the incidence of initial caries. It is also important to underline that FMR were not associated with any harmful health effects in any of the reviewed studies.

The outcome of this review was partly in contrast with a previous systematic review by Jha and co-authors [25]. Based on five papers, their narrative synthesis was in favor of FMR as a routine measure in clinical practice due to its anticipated capacity to inhibit the formation of white spot lesions and dental caries during treatment with fixed appliances. Our somewhat diverging conclusion was explained by different inclusion criteria, resulting in a limited overlap as only three primary trials were common in both reviews. Other recent systematic and umbrella reviews focused on the full spectrum of caries preventive measures [26], and included trials dealing with treatment and regression of existing post-orthodontic white spot lesions, which was out of the scoop of the present review.

The limited beneficial effects of FMR displayed in the current review merits some reflections. We assessed the effect on subject level as “no demineralization” versus “any demineralization” and not considered neither the frequency nor the severity of lesions. Thus, the undisputed caries preventive effect of topical fluorides may have been somewhat underestimated. It should also be underlined that the long-term effect of regular fluoride exposure on the oral microbiota and dental biofilm in orthodontic patients is not fully clear [27]. Furthermore, the impact of FMR on caries development may have been compromised by the general decline in caries over the years, paralleled with the introduction of modern fluoride toothpaste [28]. Today, more than 90% of the young population in high- and middle income countries report daily use of fluoride toothpaste [29, 30]. Thus, we deliberately limited the literature search back to 1985 in order to reflect ‘todays’ scenario. Another concern is that none of the included studies applied a formal continuous assessment of the compliance. It is obvious that the effect of self-applied FMR is dependent on the adherence to the study protocol and there is a risk that a single check by a questionnaire may overestimate compliance. Unfortunately, studies indicate that less than 25% of orthodontic patients actually use FMR on a regular basis [12, 31]. It was also unclear whether the participants were subjected to a formal caries risk assessment prior to the onset of the appliances. We assume that a good oral hygiene and a low caries activity are important requirements before onset of fixed orthodontic treatment, which means that children with high caries risk are rejected. As there are large socioeconomic inequalities in the burden of dental caries [32], the participants included in this review are likely not covering the entire spectrum of the population.

In this project, we included studies that scored the endpoint in different ways but we focused the development of new initial lesions. Unfortunately, we noted inconsistent ways to report the incidence of caries during treatment, varying from subject/tooth/site level, mean values of lesion area, frequency figures and raw numbers. For example, Ravikiran and co-workers [16] presented mean values of the demineralized surface area of the affected teeth. Such different modes of reporting endpoints hamper future systematic reviews and meta-analysis so we underline the importance of establishing and adhere to a core outcome set (COS) in orthodontic trials [33, 34]. In one study assessments of proximal caries incidence were made as well [18]. However, just the data on the incidence of white spot lesions was included in our meta-analysis as different endpoints for the assessments of the incidences of white spot lesions and proximal caries were reported. Moreover, none of our selected studies addressed health-economic aspects, methods to increase adherence to self-applied topical fluorides, reflected the patient’s perspectives or the perceived value of the intervention. Such information is both highly relevant and important in the process of developing guidelines and treatment recommendations.

## Conclusion

This systematic review and meta-analysis found insufficient support for a general recommendation to use FMR in order to prevent caries incidence during treatment with fixed orthodontic appliances in populations with regular use of fluoride toothpaste. This does not rule out the possibility that individual orthodontic patients may benefit from FMR after comprehensive risk assessment. Further and larger investigations reporting a core outcome set are required to clarify if this intervention should be recommended for orthodontic patients.

## Electronic supplementary material

Below is the link to the electronic supplementary material.

Supplementary Material 1

Supplementary Material 2

## Data Availability

Data is provided within the manuscript or supplementary information files.
