# Supplementary material for: Fluoride mouthrinses for prevention of initial caries in orthodontic patients - a systematic review and meta-analysis
Source: BMC Oral Health. 2025 Jul 2;25:1058. doi: 10.1186/s12903-025-06374-8 (PMC12225201; doi:10.1186/s12903-025-06374-8)
Supplement: Supplementary file 1 — Supplementary Material 1 [file 12903_2025_6374_MOESM1_ESM.docx]

**Supplementary file A. Search strings**

**PubMed via NLM September 30 2024**

|  |  |  |
| --- | --- | --- |
|  | dental white spot[tiab] OR dental white spots[tiab] OR white spot lesion[tiab] OR white spot lesions[tiab] OR initial caries[Title/Abstract] OR enamel decalcification[Title/Abstract] OR enamel demineralization[Title/Abstract] OR "Dental Caries"[Mesh:NoExp] | [52,607](https://pubmed.ncbi.nlm.nih.gov/?term=dental+white+spot%5Btiab%5D+OR+dental+white+spots%5Btiab%5D+OR+white+spot+lesion%5Btiab%5D+OR+white+spot+lesions%5Btiab%5D+OR+initial+caries%5BTitle%2FAbstract%5D+OR+enamel+decalcification%5BTitle%2FAbstract%5D+OR+enamel+demineralization%5BTitle%2FAbstract%5D+OR+%22Dental+Caries%22%5BMesh%3ANoExp%5D&sort=date&ac=no) |
|  | **fluoride[tiab] OR fluorides[tiab] OR "Fluorides"[Mesh]** | [71,037](https://pubmed.ncbi.nlm.nih.gov/?term=fluoride%5Btiab%5D+OR+fluorides%5Btiab%5D+OR+%22Fluorides%22%5BMesh%5D&sort=date&ac=no) |
|  | **mouth wash[tiab] OR mouth washes[tiab] OR mouthwash[tiab] OR mouthwashes[tiab] OR mouth rinse[tiab] OR mouth rinses[tiab] OR "Mouthwashes"[Mesh]** | [10,568](https://pubmed.ncbi.nlm.nih.gov/?term=%22Mouthwashes%22%5BMesh%5D+OR+mouth+wash%5Btiab%5D+OR+mouth+washes%5Btiab%5D+OR+mouthwash%5Btiab%5D+OR+mouthwashes%5Btiab%5D+OR+mouth+rinse%5Btiab%5D+OR+mouth+rinses%5Btiab%5D&sort=date&ac=no) |
|  | 1 AND 2 AND 3 | 866 |
|  | Limit to Jan 1 1985 | 592 |

Cochrane via Wiley

|  |  |  |
| --- | --- | --- |
|  | MeSH descriptor: [Dental Caries] this term only OR (dental white spot OR dental white spots OR white spot lesion OR white spot lesions OR OR initial caries OR enamel decalcification OR enamel demineralization):ti,ab,kw (Word variations have been searched) | 5,099 |
|  | **(fluoride):ti,ab,kw (Word variations have been searched) OR MeSH descriptor: [Fluorides] explode all trees** | 6,787 |
|  | **(mouthwashes OR mouth washes OR mouth rinses):ti,ab,kw (Word variations have been searched) OR MeSH descriptor: [Mouthwashes] explode all trees** | 6,110 |
|  | 1 AND 2 AND 3 | 240 |

Web of Science via Clarivate

|  |  |  |
| --- | --- | --- |
|  | **TS=("dental white spot" OR "dental white spots" OR "white spot lesion" OR "white spot lesions"OR "initial caries" OR "enamel decalcification" OR "enamel demineralization")** | 2,858 |
|  | TS=(fluoride OR fluorides) | 158,073 |
|  | **TS=(mouthwashes OR "mouth washes" OR "mouth wash" OR "mouth rinse" OR "mouth rinses")** | 5,884 |
|  | 1 AND 2 AND 3 | 53 |

Scopus via Elsevier

|  |  |  |
| --- | --- | --- |
|  | TITLE-ABS-KEY("dental white spot" OR "dental white spots" OR "white spot lesion" OR "white spot lesions"OR "initial caries" OR "enamel decalcification" OR "enamel demineralization") | 12,201 |
|  | TITLE-ABS-KEY ( fluoride OR fluorides ) | 200,379 |
|  | TITLE-ABS-KEY ( mouthwashes OR "mouth washes" OR "mouth wash" OR "mouth rinse" OR "mouth rinses" ) | 12,201 |
|  | 1 AND 2 AND 3 | 88 |

Google Scholar

|  |  |  |
| --- | --- | --- |
|  | “dental white spot*” “fluor* “mouthwash”  "white spot lesion*" fluor* "mouthwash"  "initial caries" fluor* "mouthwash"  "initial caries" fluor* "mouth rinse*" | 178 |
|  | Limit to 1 jan 1985 | 173 |

Searched 5-10 pages
