# Supplementary material for: Fluoride mouthrinses for prevention of initial caries in orthodontic patients - a systematic review and meta-analysis
Source: BMC Oral Health. 2025 Jul 2;25:1058. doi: 10.1186/s12903-025-06374-8 (PMC12225201; doi:10.1186/s12903-025-06374-8)
Supplement: Supplementary file 2 — Supplementary Material 2 [file 12903_2025_6374_MOESM2_ESM.docx]

**Supplementary file B.** Excluded studies

_______________________________________________________________________________________

First author, year main reason for exclusion

_______________________________________________________________________________________

Boyd, 1992 Not a randomized controlled trial

Boyd, 1993 Double publication of Boyd, 1992

Dehghani, 2015 Non-eligible endpoint

Geiger, 1988 Historic control group

Geiger, 1992 No adequate control group

Hosseinpour-Nader, 2022 Intervention and follow-up was less than 6 months

Koopman, 2015 Non-eligible endpoint; same study group as van der Kaaij et al. 2015

Mishra, 2023 Post-orthodontic intervention, non-eligible endpoint

Øgaard, 2006 Two different fluoride mouthrinses were compared, no adequate control group

Pilli, 2022 Two different fluoride mouthrinses were compared, no adequate control group

Rashid, 2022 Vital information is lacking

Ravi Kiran, 2023 Double publication of Ravikiran et al. 2021

Shimpo, 2022 Fluoride gel intervention

Willmot, 2024 Post-orthodontic intervention, non-eligible endpoint

Zingler, 2016 Removable appliances, non-eligible endpoint

_______________________________________________________________________________________

**References**

Boyd RL. Two-year longitudinal study of a peroxide-fluoride rinse on decalcification in adolescent orthodontic patients. J Clin Dent. 1992;3:83-87. PMID: 1449617

Boyd RL. Comparison of three self-applied topical fluoride preparations for control of decalcification. Angle Orthod. 1993;63:25-30. [https://doi.org/10.1043/0003-3219(1993)063<0025:COTSTF>2.0.CO;2](https://doi.org/10.1043/0003-3219(1993)063%3c0025:COTSTF%3e2.0.CO;2)

Dehghani M, Abtahi M, Sadeghian H, Shafaee H, Tanbakuchi B. Combined chlorhexidine-sodiumfluoride mouthrinse for orthodontic patients: Clinical and microbiological study. J Clin Exp Dent. 2015;7:e569-575. <https://doi.org/10.4317/jced.51979>.

Geiger AM, Gorelick, L, Gwinnett AJ, Griswold PG. The effect of a fluoride program on white spot formation during orthodontic treatment. Am J Orthod Dentofacial Orthop. 1988;93:29-37. <https://doi.org/10.1016/0889-5406(88)90190-4>.

Geiger AM, Gorelick L, Gwinnett AJ, Benson BJ. Reducing white spot lesions in orthodontic populations with fluoride rinsing. Am J Orthod Dentofacial Orthop. 1992;101:403–07.

Hosseinpour-Nader A, Karimi N, Ghafari HA, Ghorbanzadeh R. Effect of nanomicelle curcumin-based photodynamic therapy on the dynamics of white spot lesions and virulence of Streptococcus mutans in patients undergoing fixed orthodontic treatment: A randomized double-blind clinical trial. Photodiagnosis Photodyn Ther. 2022;40:103183. <https://doi.org/10.1016/j.pdpdt.2022.103183>.

Koopman JE, van der Kaaij NC, Buijs MJ, Elyassi Y, van der Veen MH, Crielaard W, Ten Cate JM, Zaura E. The effect of fixed orthodontic appliances and fluoride mouthwash on the oral microbiome of adolescents – A randomized controlled clinical trial. PLoS One. 2015;10:e0137318. <https://doi.org/10.1371/journal.pone.0137318>.

Mishra S, Mani S, Sonawane A, Viragi P, Toshiwal NG, Manerikar R. Assessment of white spot lesion and enamel demineralization in orthodontic patients with fixed brackets—A clinical appraisal using fluoride, mouth rinse, fluoride varnish containing CPP-ACP, and CPP-ACFP. J Ind Orthod Soc. 2023;57:91–7. <https://doi.org/10.1177/03015742221076915>.

Øgaard B, Alm AA, Larsson E, Adolfsson U. A prospective, randomized clinical study on the effects of an amine fluoride/stannous fluoride toothpaste/mouthrinse on plaque, gingivitis and initial caries lesion development in orthodontic patients. Eur J Orthod. 2006;28:8-12. <https://doi.org/10.1093/ejo/cji075>.

Rashid MW, Hameed M, Parvez A, Mujtaba M, Nawadat K, Saqib S. Is high fluoride toothpaste or fluoride mouthwash more effective in enhancing cariostatic activity among patients of orthodontic treatment? Pakistan J Med & Health Sci. 2022;16:968-9. <https://doi.org/10.53350/pjmhs22165968>.

Ravi Kiran KR, Sabrish S, Mathew S, Shivamurthy PG, Sagarkar R. Effectiveness of amine fluoride mouthwash in preventing white spot lesions during fixed orthodontic therapy - A randomized control trial. Indian J Dent Res. 2023;34:261-5. <https://doi.org/10.4103/ijdr.ijdr_984_21>.

Pilli LN, Singaraju GS, Nettam V, Keerthipati T, Mandava P, Marya A. An extensive comparison of the clinical efficiency of acidulated phosphate fluoride (APF) and neutral sodium fluoride (NaF) oral rinses in the prevention of white spot lesions during fixed orthodontic treatment: A randomized controlled trial. Biomed Res Int. 2022;2022:6828657. <https://doi.org/10.1155/2022/6828657>.

Shimpo Y, Nomura Y, Sekiya T, Arai C, Okada A, Sogabe K, Hanada N, Tomonari H. Effects of the dental caries preventive procedure on the white spot lesions during orthodontic treatment-An open label randomized controlled trial. J Clin Med. 2022;11:854. <https://doi.org/10.3390/jcm11030854>.

Willmot DR. White lesions after orthodontic treatment: does low fluoride make a difference? J Orthod. 2004;31:235-42; discussion 202. <https://doi.org/10.1179/146531204225022443>.

Zingler S, Pritsch M, Lux CJ, Kneist S. Association between clinical and salivary microbial parameters during orthodontic treatment with removable appliances with or without use of fluoride mouth rinse. Eur J Paediatr Dent. 2016;17:181-7.
